# Author Correction: Engineering immunomodulatory and osteoinductive implant surfaces via mussel adhesion-mediated ion coordination and molecular clicking

**DOI:** 10.1038/s41467-026-77695-7

**Published:** 2026-09-09

**Authors:** Tao Wang, Jiaxiang Bai, Min Lu, Chenglong Huang, Dechun Geng, Gang Chen, Lei Wang, Jin Qi, Wenguo Cui, Lianfu Deng

**Affiliations:** 1 Department of Orthopaedics, Shanghai Key Laboratory for Prevention and Treatment of Bone and Joint Diseases, Shanghai Institute of Traumatology and Orthopaedics, Ruijin Hospital, Shanghai Jiao Tong University School of Medicine, 197 Ruijin 2nd Road, 200025 Shanghai, P. R. China; 2 Department of Orthopaedics, Shanghai General Hospital, Shanghai Jiao Tong University School of Medicine, 85 Wujin Road, 200080 Shanghai, P. R. China; 3 Jiaxing Key Laboratory of Basic Research and Clinical Translation on Orthopedic Biomaterials, Department of Orthopaedics, The second Affiliated Hospital of Jiaxing University, 1518 North Huancheng Road, 314000 Jiaxing, P. R. China; 4 Department of Orthopaedics, The First Affiliated Hospital of Soochow University, 188 Shizi Street, Suzhou, 215006 Jiangsu P. R. China

**Keywords:** Implants, Chemical engineering, Bioinspired materials

Correction to: *Nature Communications* https://doi.org/10.1038/s41467-021-27816-1, published online 10 January 2022

In the originally published version of this article, the N1s spectra presented in Fig. 2N were inadvertently duplicated from the N1s spectra of BMP-2 and Zn/BMP-2 shown in Fig. 2k. The affected spectra in Fig. 2N have been replaced with the corrected spectra, and the corresponding updated data have been provided.

The raw data file associated with Supplementary Fig. 15 was uploaded incorrectly. The Supplementary Fig. 15 itself was correct, and the issue was limited to the associated dataset file. The originally submitted dataset has been replaced with the corrected file.

These corrections do not affect the interpretation of the results or the conclusions of the article. For comparison, the original Fig. 2 is available as Supplementary Information accompanying this amendment.

## Supplementary information

Original Fig. 2

