# Supplementary material for: Engineering immunomodulatory and osteoinductive implant surfaces via mussel adhesion-mediated ion coordination and molecular clicking
Source: Nat Commun. 2022 Jan 10;13:160. doi: 10.1038/s41467-021-27816-1 (PMC8748715; doi:10.1038/s41467-021-27816-1)
Supplement: Supplementary file 1 — Supplementary information [file 41467_2021_27816_MOESM1_ESM.docx]

**Engineering Immunomodulatory and Osteoinductive Implant Surfaces via** **Mussel Adhesion-Mediated Ion Coordination and Molecular Clicking**

*Tao Wang^1,2 ,3^*†*, Jiaxiang Bai^4^*†*, Min Lu^1^, Chenglong Huang^3^, Dechun Geng^4^, Gang Chen^3^, Lei Wang^1^, Jin Qi^1^***, Wenguo Cui^1^***, and Lianfu Deng^1^**

^1^Department of Orthopaedics, Shanghai Key Laboratory for Prevention and Treatment of Bone and Joint Diseases, Shanghai Institute of Traumatology and Orthopaedics, Ruijin Hospital, Shanghai Jiao Tong University School of Medicine, 197 Ruijin 2nd Road, Shanghai 200025, P. R. China.

^2^Department of Orthopaedics，Shanghai General Hospital, Shanghai Jiao Tong University School of Medicine, 85 Wujin Road, Shanghai 200080, P. R. China

^3^Jiaxing Key Laboratory of Basic Research and Clinical Translation on Orthopedic Biomaterials, Department of Orthopaedics, The second Affiliated Hospital of Jiaxing University, 1518 North Huancheng Road, Jiaxing 314000, P. R. China.

^4^Department of Orthopaedics, The First Affiliated Hospital of Soochow University, 188 Shizi Street, Suzhou, Jiangsu 215006, P. R. China.

†These authors contributed equally to this work.

*Corresponding author. E-mail: jinjin838@hotmail.com (J. Qi); [wgcui80@hotmail.com](mailto:wgcui80@hotmail.com) (W. Cui); [lf_deng@126.com](mailto:lf_deng@126.com) (L. Deng)

***
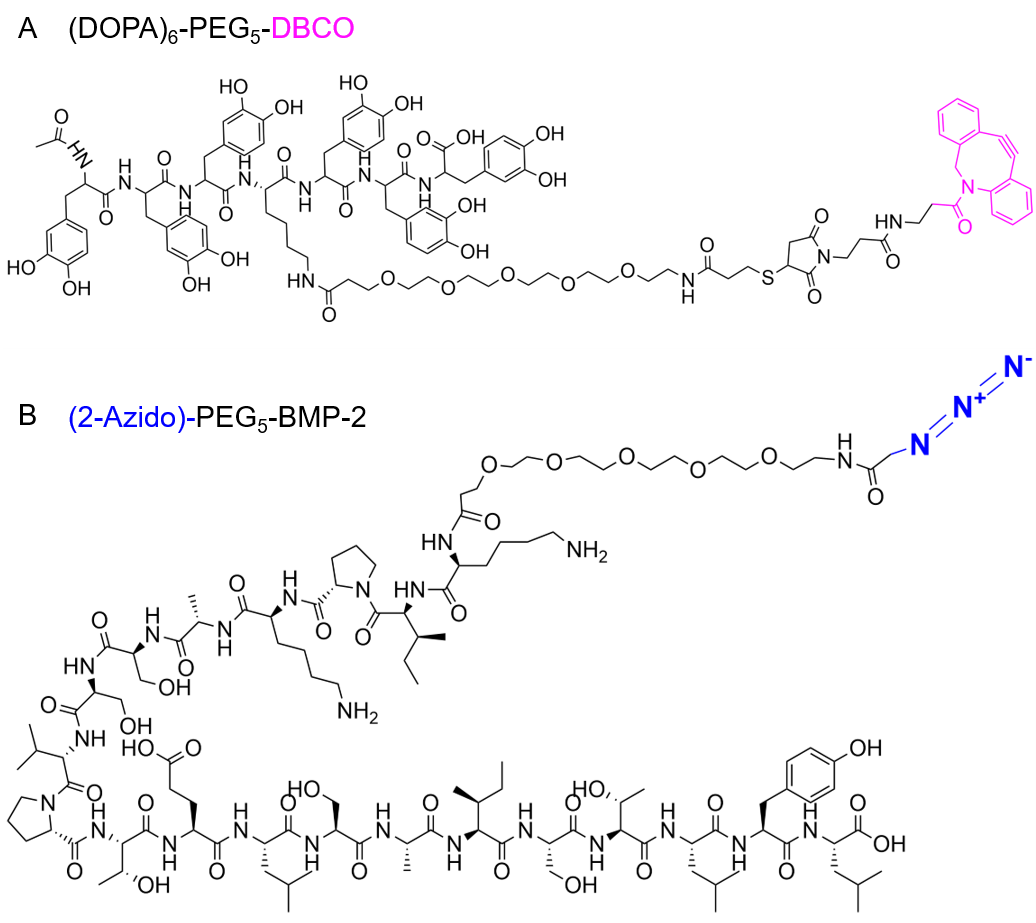
***

Supplementary ***Fig.1*** *The molecular structures of (DOPA)_6_-PEG_5_-DBCO (A) and (2-Azido)-PEG_5_-BMP-2 (B).*

***
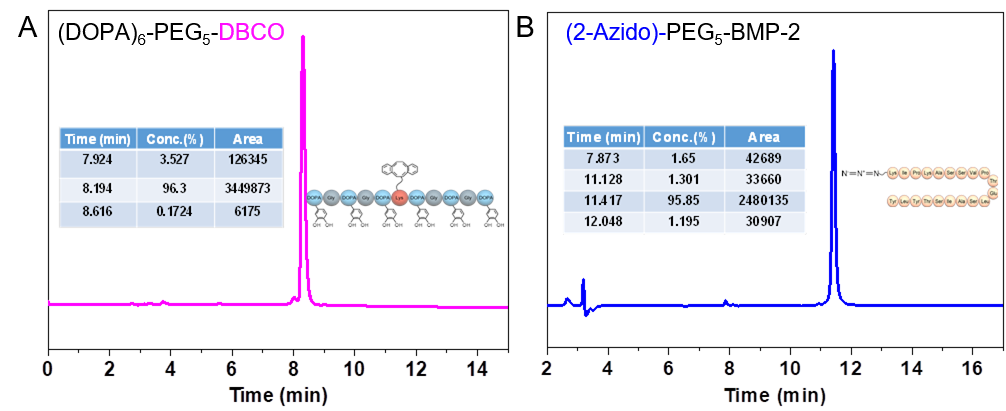
***

***Supplementary Fig.2*** *HPLC spectra of (DOPA)_6_-PEG_5_-DBCO (A) and (**2-Azido)-PEG_5_-BMP-2 (B) with purity (>95%).*

***
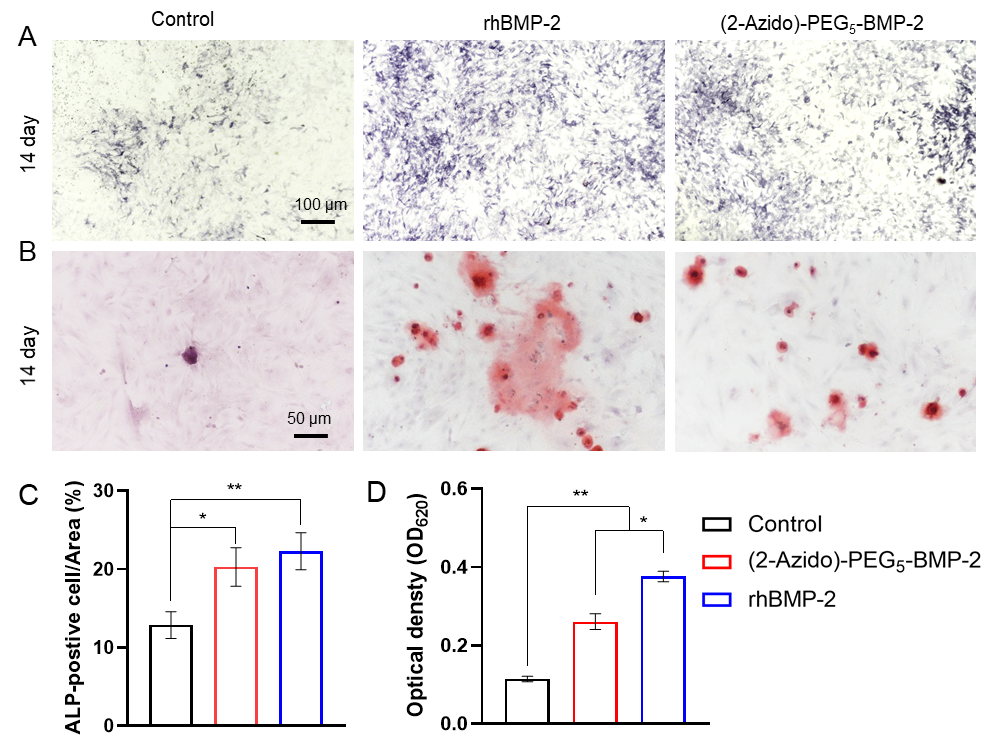
***

***Supplementary Fig.3*** *(A, C)* *ALP staining and (B, D) Alizarin Red S staining of BM-MSCs cultured in osteogenic medium supplemented with rhBMP-2 or (2-Azido)-PEG_5_-BMP-2 (50 ng/ml) (C-D, n=3 biologically independent samples per group, by a one-way ANOVA with a Tukey’s post hoc test* *for multiple comparisons). Data are reported as mean ± SD, * p<0.05, ** p<0.01).*

***
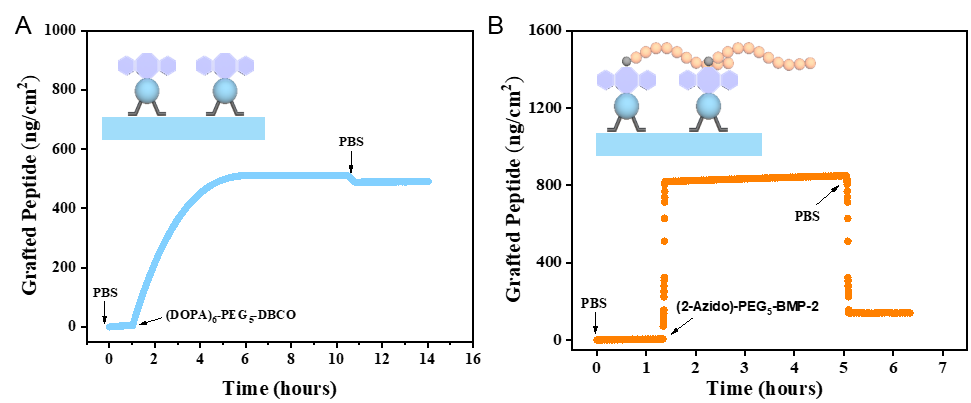
***

***Supplementary Fig.4*** *(A) Real-time monitoring of the binding of (DOPA)_6_-PEG_5_-DBCO on a TiO_2_-coated chip determined by QCM. (B) (2-Azido)-PEG_5_-BMP-2 co-grafting process on the (DOPA)_6_-PEG_5_-DBCO-bound chips.*

***
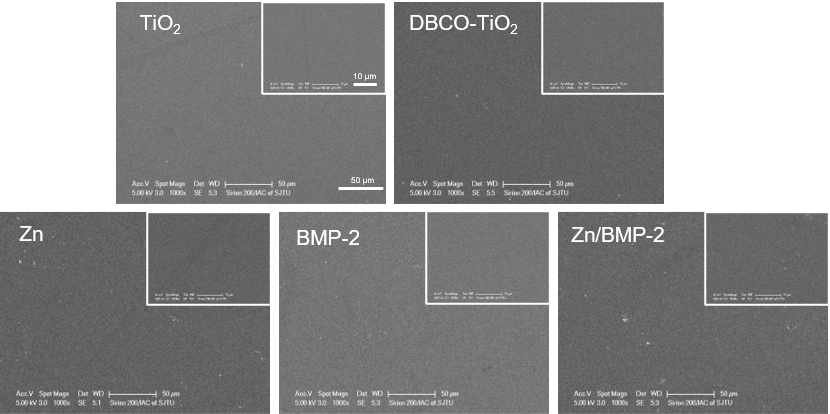
***

***Supplementary Fig.5*** *SEM images of the different surfaces (TiO_2_, DBCO-TiO_2_, Zn, BMP-2 and Zn/BMP-2 groups, three independent experiments).*

*
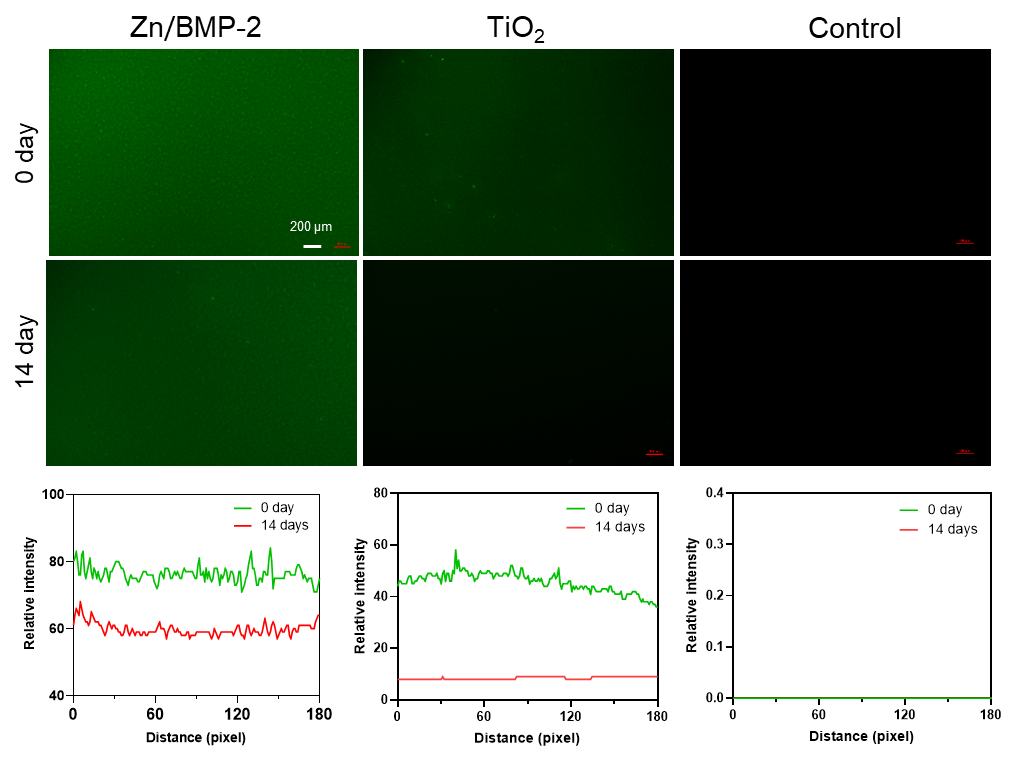
*

***Supplementary Fig.6*** *(A) Images of FITC-**(2-Azido)-PEG_5_-BMP-2 and Zn^2+^ co-modified surfaces:(green: (2-Azido)-PEG_5_-BMP-2(three independent experiments) and (B) Intensity profile with regions of interest.*

*
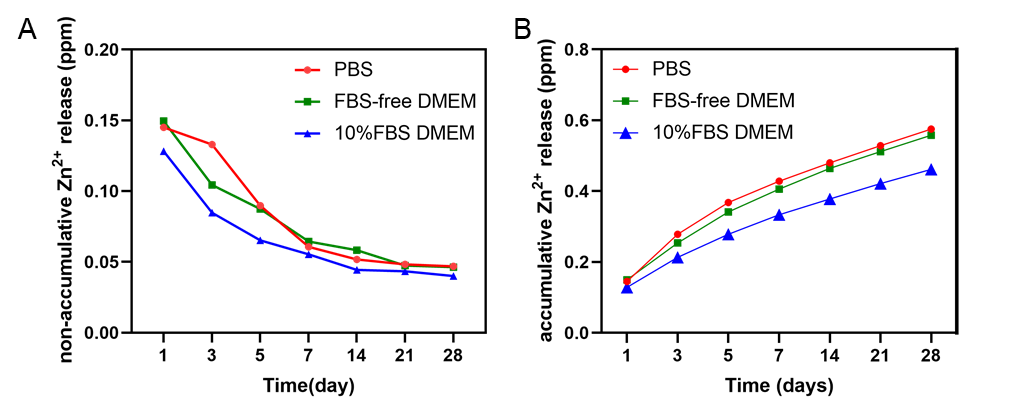
*

***Supplementary Fig.7*** *Zn^2+^ release profiles of the Zn/BMP-2 surface in PBS solution, FBS-free DMEM and 10% FBS-DMEM. (A) non-accumulative Zn^2+^ release; (B) accumulative Zn^2+^ release.*

*
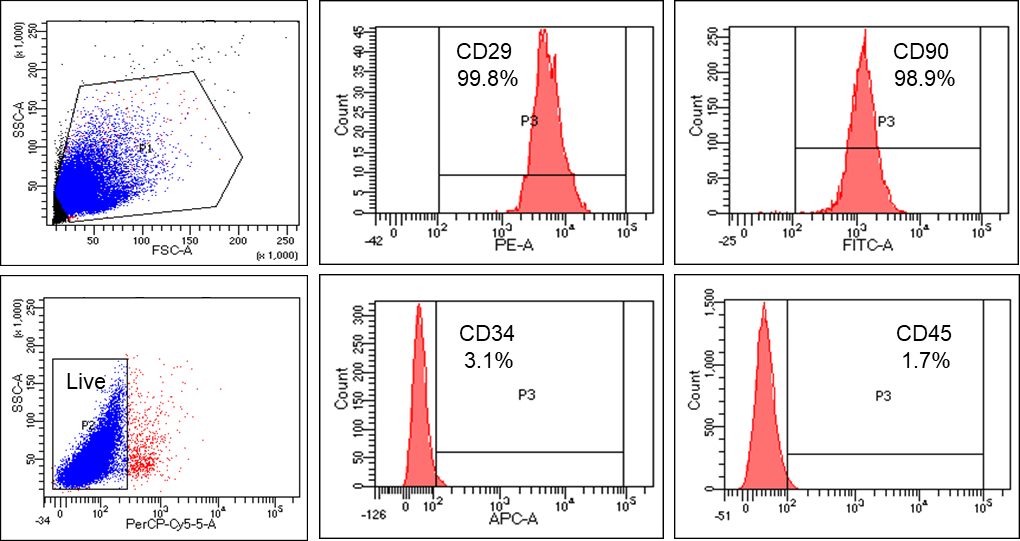
*

***Supplementary Fig.8*** *Immunophenotypic characterization of BM-MSCs. Gating strategies for flow cytometry data: the total BM-MSC cells were first gated by forward and side scatter, PerCp-Cy5.5 negative cells were further gated as live cells to identify the expression of surface markers of CD29 CD34 CD45. FACS results showed that these cells were homogenously positive for mesenchymal markers CD29 and CD90; negative for hematopoietic markers CD34 and CD45* *(n=3, independent samples per group).*

*
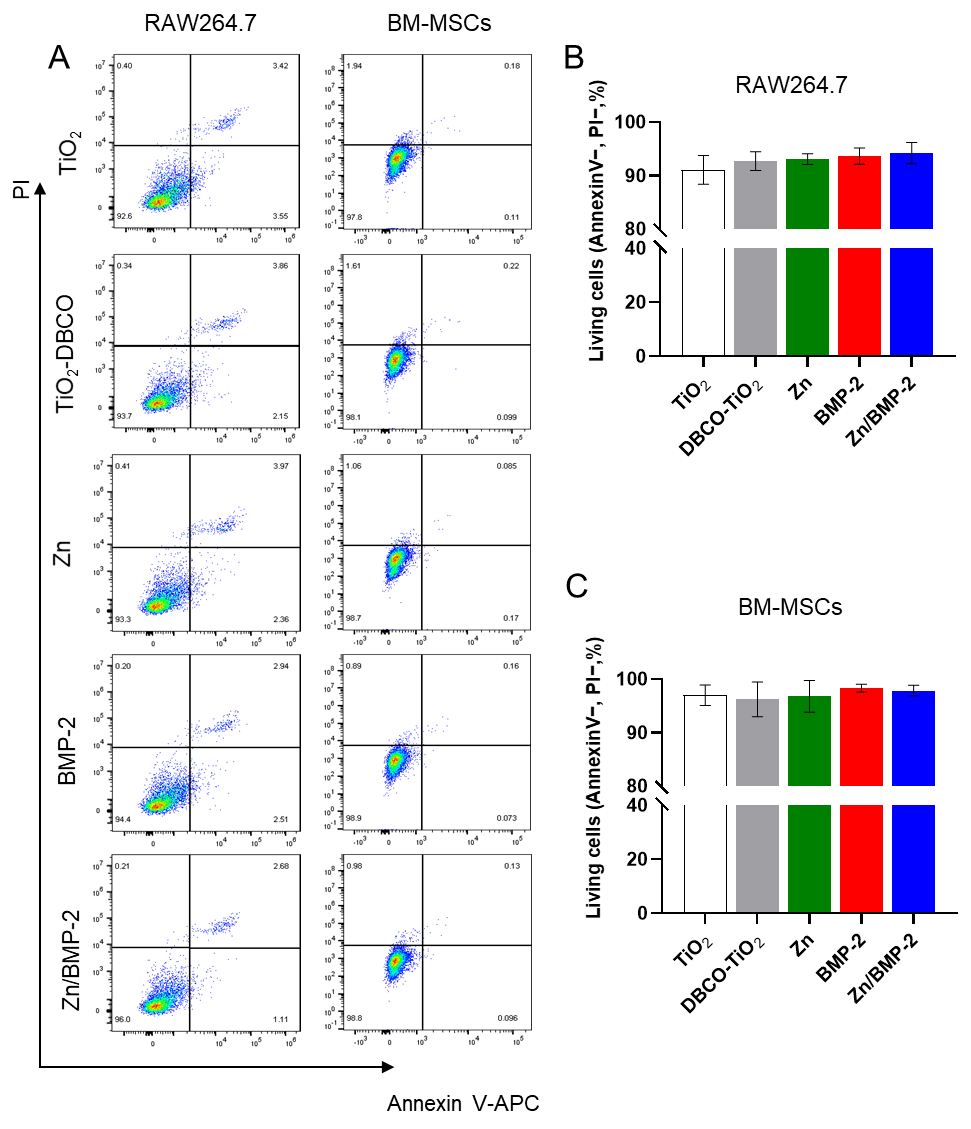
*

***Supplementary Fig.9*** *Percentages of living RAW264.7 and BM-MSCs cultured on samples using Annexin V-PI staining determined by flow cytometry (****B-C,*** *n=3 biologically independent samples per group, by a one-way ANOVA with a Tukey’s post hoc test* *for multiple comparisons).* *Data are reported as mean ± SD,* ** p<0.05, ** p<0.01. No statistical significance has been found between groups.*

*
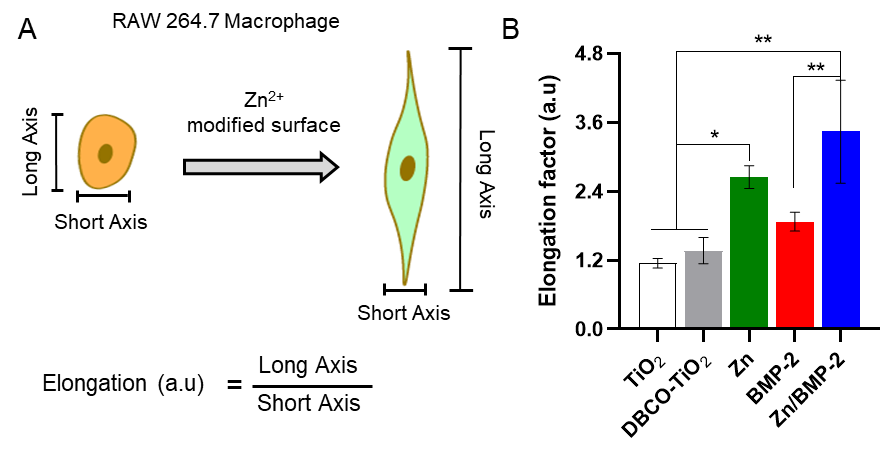
*

***Supplementary Fig.10*** *(A) Scheme of cell morphology switch and definition of elongation factor; (B) Quantitative data of RAW264.7 elongation factor (B, n=3 biologically independent samples per group, by a one-way ANOVA with a Tukey’s post hoc test for multiple comparisons). Data are reported as mean ± SD, * p<0.05, ** p<0.01.*

***
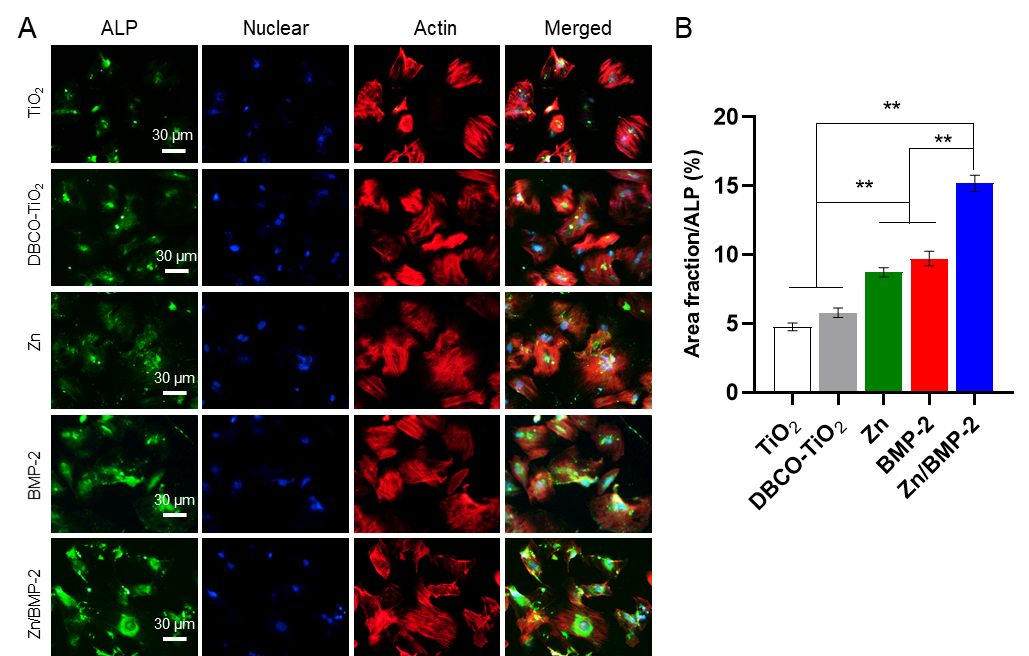
***

***Supplementary Fig.11*** *Images of the BM-MSCs after immunofluorescent staining:(green: ALP; red: cytoskeleton and blue: nuclei) and (B) quantitative results (B, n=3 biologically independent samples per group, by a one-way ANOVA with a Tukey’s post hoc test* *for multiple comparisons). Data are reported as mean ± SD, * p<0.05, ** p<0.01.*


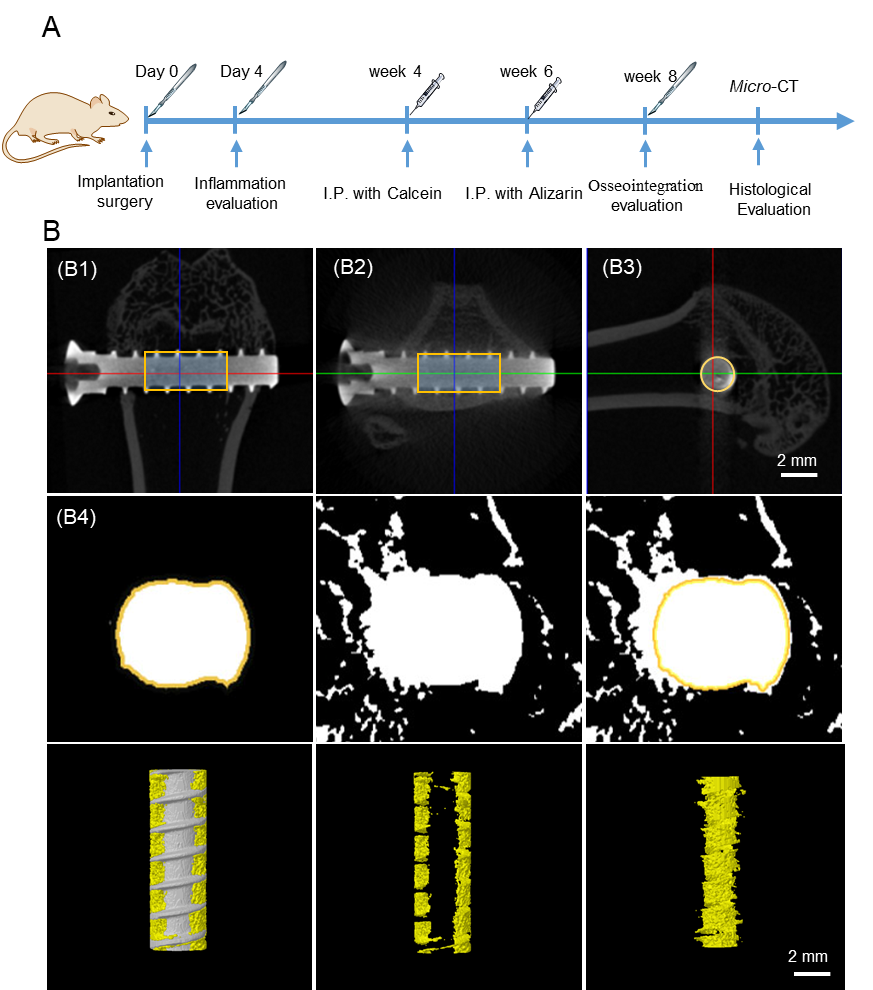


***Supplementary Fig.12 (A)*** *Scheme for implantation surgery for in vivo tests and treatment process;* ***(B)*** *At each 3D location (shown as cross-hair), three orthogonal views are retrieved from the whole dataset and shown as (B1) coronal view (the normal images, in x-y plane), (B2) transaxial view (x-z plane), (B3) sagittal view (z-y plane); (B4)* *Upper: Semi-automated image segmentation was used to define the boundary where new bone occurs (yellow) on a 2D tomogram. The volume of interest is outside the boundary; Lower: 3D rendering of the entire volume of interest and a corresponding longitudinal cut-away view for a representative specimen.*

*
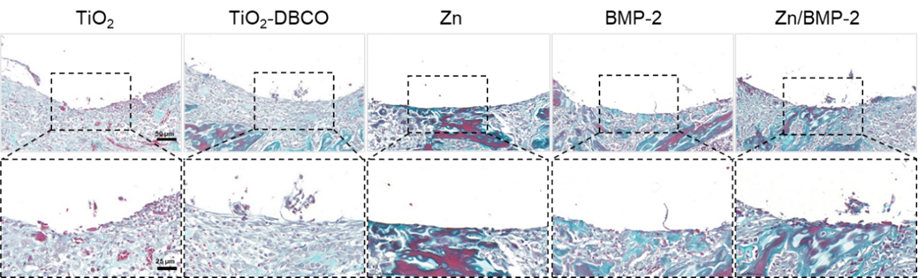
*

***Supplementary Fig.13*** *Goldner’s trichrome staining for the tissue around bone implants (n=3, independent samples per group).*

***
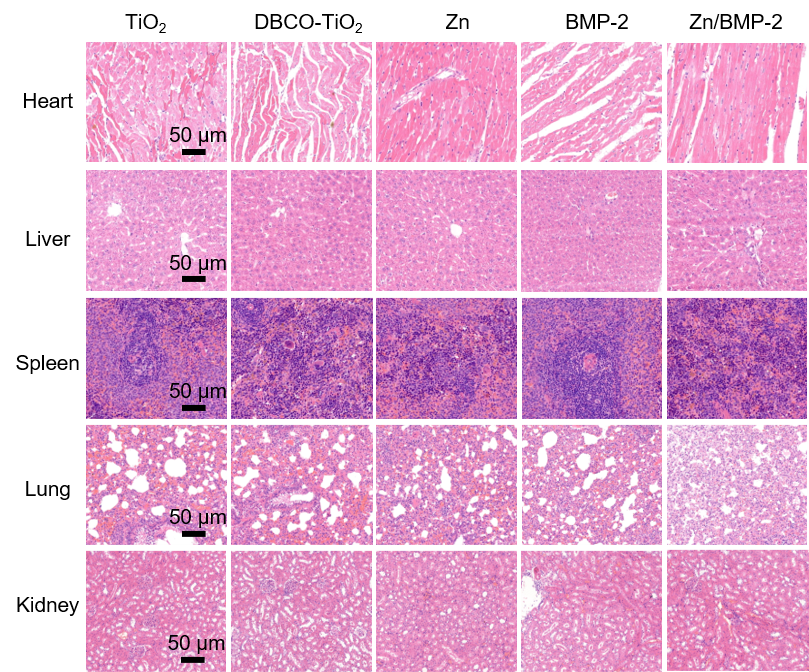
***

***Supplementary Fig.14*** *Toxicities of samples on heart, liver, spleen, lung and kidney. H&E staining of the organ tissue sections (three independent experiments, scar bar=50 μm).*

***
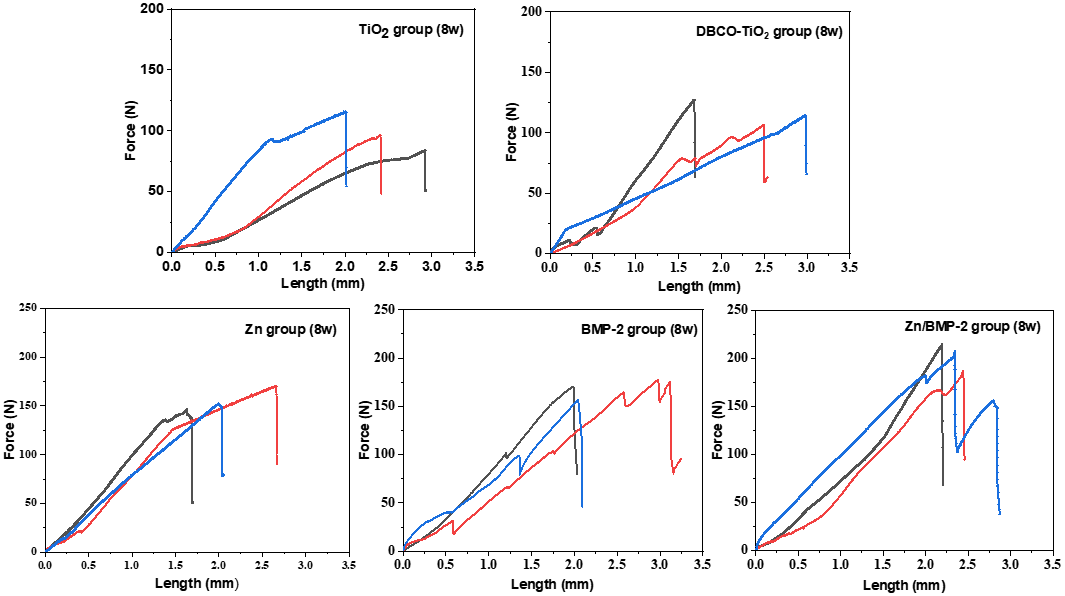
***

***Supplementary Fig.15*** *Biomechanical pull-out testing curves of different peptide-treated and untreated Ti screws (n=3, independent sample per group).*

**Supplementary Table 1*.*** Statistics of chemical compositions using XPS on the different surfaces *(TiO_2_, DBCO-TiO_2_, Zn, BMP-2 and Zn/BMP-2 groups).*

| Element | Atomic (%) | | | | |
| --- | --- | --- | --- | --- | --- |
|  | TiO_2_ | DBCO-TiO_2_ | Zn | BMP-2 | Zn/BMP-2 |
| Ti | 16.71 | 12.65 | 10.0 | 9.83 | 9.15 |
| C | 39.22 | 45.97 | 44.77 | 50.30 | 45.10 |
| O | 43.20 | 37.15 | 39.73 | 33.10 | 38.29 |
| N | 0.87 | 4.20 | 2.72 | 6.74 | 4.89 |
| Zn | 0.00 | 0.00 | 2.78 | 0.03 | 2.57 |
| N/Ti | 0.052 | 0.332 | 0.272 | 0.686 | 0.534 |
| Zn/Ti | 0.000 | 0.000 | 0.278 | 0.003 | 0.281 |

**Supplementary Table 2*.*** *Primers used in the RT-PCR of BM-MSCs and BMMs cells.*

| Cell | Gene | Primers Sequence (5‘-3‘) |
| --- | --- | --- |
| BM-MSCs | *Alp* | F: ATGCTCAGGACAGGATCAAA |
|  |  | R: CGGGACATAAGCGAGTTTCT |
|  | *Col1a1* | F: AGCTCGATACACAATGGCCT |
|  |  | R: CCTATGACTTCTGCGTCTGG |
|  | *Runx2* | F: ATCATTCAGTGACACCACCA |
|  |  | R: GTAGGGGCTAAAGGCAAAAG |
|  | *Opn* | F: GAACATGAAATGCTTCTTTCTCAG |
|  |  | R: TCCATGAAGCCACAAACTAAACTA |
|  | *β-actin* | F: CCTCTATGACAACACAGT |
|  |  | R: AGCCACCAATCCACACAG |
| RAW264.7 | *Tnf-α* | F: GTTCCCAAATGGCCTCCC |
|  |  | R: GTGCTCCTCACCCACACCG |
|  | *Il10* | F: CCCTTTGCTATGGTGTCCT |
|  |  | R: GTGGCCAGTTTGTTATTTAT |
|  | *Cd206* | F: TACTTGGACGGATAGATGGAGG |
|  |  | R: CATAGAAAGGAATCCACGCAGT |
|  | *Ccr7* | F: GGTGGCTCTCCTTGTCATTTTC |
|  |  | R: AGGTTGAGCAGGTAGGTATCCG |
|  | *Vegf* | F: AGGAGTCCCCGACGAGATAGA |
|  |  | R: CACATCTGCTGTGCTGTAGGAA |
|  | *Bmp-2* | F: AACGAGAAAAGCGTCAAGCC |
|  |  | R: AGGTGCCACGATCCAGTCAT |
|  | *β-actin* | F: GTGACGTTGACATCCGTAAAGA |
|  |  | R: GTAACAGTCCGCCTAGAAGCAC |
