# Supplementary material for: Author Correction: Engineering immunomodulatory and osteoinductive implant surfaces via mussel adhesion-mediated ion coordination and molecular clicking
Source: Nat Commun. 2026 Sep 9;17:9650. doi: 10.1038/s41467-026-77695-7 (PMC13558590; doi:10.1038/s41467-026-77695-7)
Supplement: Supplementary file 1 [file 41467_2026_77695_MOESM1_ESM.pdf]

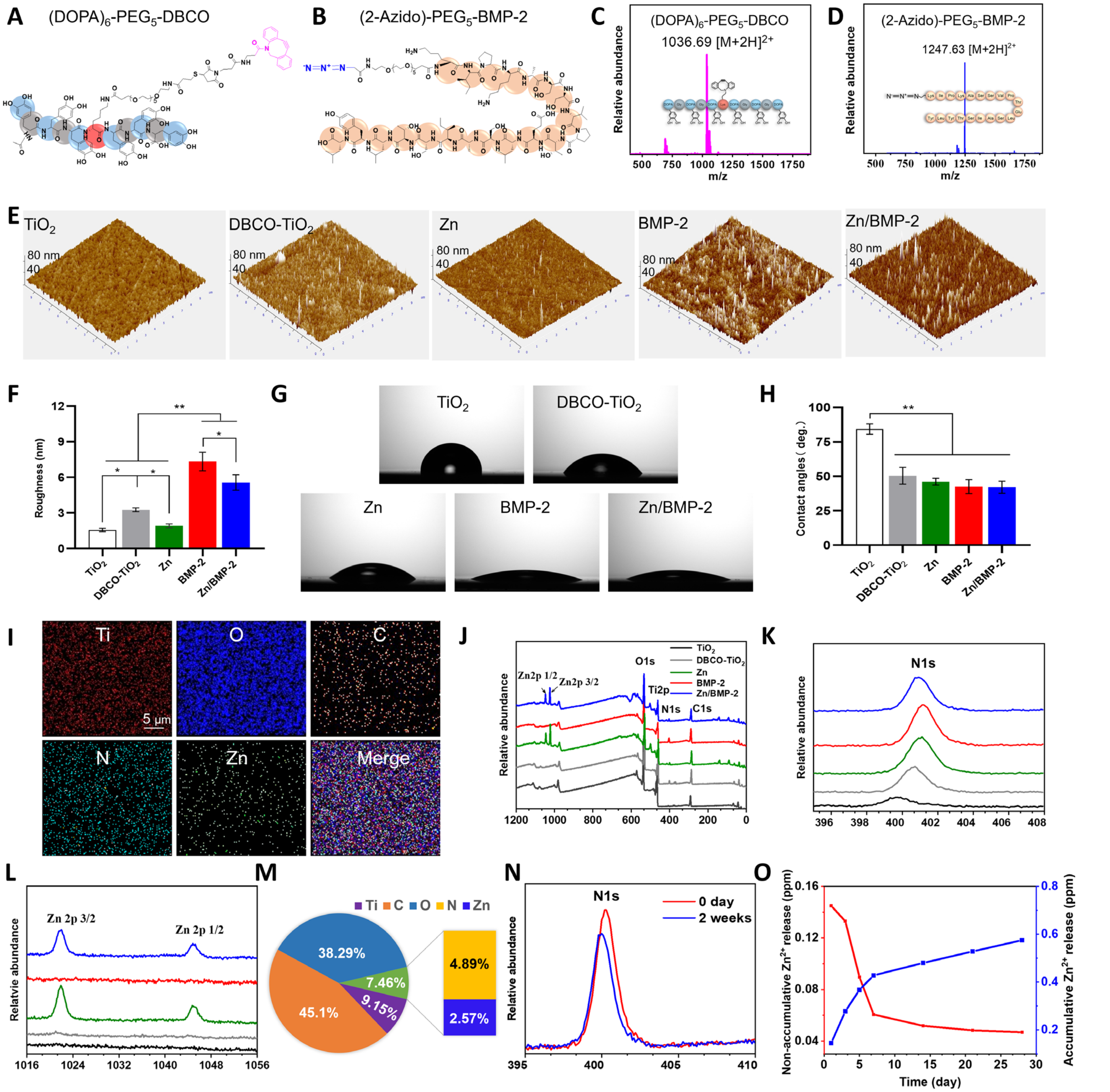

Fig. 2 Characterizations of clickable peptide and Zn^{2+} and BMP-2 peptide co-modified surface. A, **B** The molecular structures of $(DOPA)_6$-PEG_5-DBCO and (2-Azido)-PEG_5-BMP-2. **C**, **D** ESI-MS spectra of the two synthetic peptides. **E** AFM images of different surfaces and (**F**) quantitation of the suface roughness with the different modified surfaces ($n = 3$ independent samples per group, by a one-way ANOVA with a Tukey's post hoc test for multiple comparisons). Data are reported as mean ± SD, $*p < 0.05$, $**p < 0.01$). **G** The water contact angles of different surfaces and (**H**) the quantitative results ($n = 3$ independent samples per group, by a one-way ANOVA with a Tukey's post hoc test for multiple comparisons. Data are reported as mean ± SD, $**p < 0.01$). **I** SEM-EDS elemental mapping for the Zn^{2+} and BMP-2 peptide co-modified surface (Zn/BMP-2) (scar bar = 5 μm, three independent experiments). **J**–**L** XPS analysis of the bare and modified TiO_2 surface (DBCO-TiO_2, Zn, BMP-2 and Zn/BMP-2. **M** Quantitative elemental analysis according to XPS. **N** Changes of N 1*s* signal in the XPS spectrum of the Zn/BMP-2 surface after incubated in DMEM for 2 weeks. **O** Zn^{2+} release profiles of the Zn/BMP-^{2} surface in PBS solution; red (left) and blue (right) represent the non-accumulative and accumulative Zn^{2+} release, respectively. Exact *P* values were given in the Source Data file.
